# Dysregulation of bladder corticotropin-releasing hormone receptor in the pathogenesis of human interstitial cystitis/bladder pain syndrome

**DOI:** 10.1038/s41598-019-55584-y

**Published:** 2019-12-16

**Authors:** Jia-Fong Jhang, Lori A. Birder, Yuan-Hong Jiang, Yung-Hsiang Hsu, Han-Chen Ho, Hann-Chorng Kuo

**Affiliations:** 1 Department of Urology, Hualien Tzu Chi Hospital, Buddhist Tzu Chi Medical Foundation, Hualien, Taiwan, Hualien, Taiwan; 2 Departments of Medicine; Pharmacology and Chemical Biology, University of Pittsburgh, Pittsburgh, Pennsylvania USA; 3 Department of Pathology, Hualien Tzu Chi Hospital, Buddhist Tzu Chi Medical Foundation, Hualien, Taiwan, Hualien, Taiwan; 4 Department of Anatomy, Tzu Chi University, Hualien, Taiwan

**Keywords:** Bladder disease, Chronic inflammation

## Abstract

Stress is associated with exacerbated symptoms in patients with interstitial cystitis/bladder pain syndrome (IC/BPS). To investigate the mechanism of stress implicated on IC/BPS, we investigated expression of stress-response receptor corticotropin-releasing hormone receptor (CRHR) in bladder from IC/BPS patients. Twenty-three IC/BPS patients with Hunner’s lesion (HIC), 51 IC/BPS patients without Hunner’s lesion (NHIC), and 24 patients with stress urinary incontinence as controls were enrolled. Cystoscopic biopsies of bladder wall including mucosa and submucosa were obtained from all patients. Western blotting was used to investigate the bladder expression of the CRHR1 and CRHR2. Immunochemical staining revealed CRHR1 expression was mainly located in the submucosa while CRHR2 expression was mainly in uroepithelial cells. Compared to control subjects, the CRHR1 expression was significantly higher, while CRHR2 expression was significantly lower in IC/BPS patients. Further analysis of patients with HIC, NHIC, and control subjects showed that bladder in patients with HIC had significantly higher expressions of CRHR1 and significantly lower CRHR2. CRHR2 expression was significantly negatively correlated with O’Leary-Sant score and bladder pain. Our results indicate dysregulation of bladder CRHR1 and CRHR2 in patients with IC/BPS, and suggest CRH signaling may be associated with IC/BPS symptoms.

## Introduction

Interstitial cystitis/bladder pain syndrome (IC/BPS)is a heterogeneous syndrome that is diagnosed on the basis of an unpleasant sensation perceived to be related to the urinary bladder and associated with lower urinary tract symptoms^1^. The etiology of IC/BPS is poorly understood, and it may involve multiple pathways leading to variable clinical symptoms. Recent studies revealed urothelial function abnormality in the pathogenesis of IC/BPS involving several possible mechanisms^2^. Upregulation of purinergic receptor P2X3 and decreased muscarinic receptors M3 in the urothelium has been identified in previous IC/BPS studies^3,4^. Overexpression of multiple factors including nerve growth factor (NGF) has been reported in the urothelium of a naturally occurring model of IC in cats termed “FIC”^5^. There is considerable evidence that changes in urothelial targets and signaling mechanism may somehow play an important role in sensory dysfunction in IC/BPS.

Most patients with IC/BPS recognize that daily stress plays a part in exacerbating symptoms which can result in a pain flare. In a prospective study, significant relationships between stress and bladder pain, urgency, and nocturia were observed in patients with IC/BPS^6^. Recently, many animal studies also investigated the role of chronic stress in the pathogenesis of IC/BPS. For example, rats exposure to chronic psychological stress (water avoidance stress or WAS) result in a visceral hyperalgesia and increased numbers of mast cells in the mucosa^7^. Rats exposed to WAS demonstrated increased voiding frequency, and this behavioral has also been found to be correlated with decreases in spinal glutamate levels^8^.

Corticotropin-releasing hormone (CRH) is a peptide hormone that is secreted by the paraventricular nucleus of the hypothalamus in response to stress. Hyperactivity of CRH neuronal systems is well known as a biomarker for depression and anxiety disorders^9^. Peripheral CRH signaling also plays an important role in mediating stress-induced effects on visceral organs such as those in the gastrointestinal system^9^. Urocortin (UCN) is a member of the CRH neuropeptide family and has a high affinity to peripheral CRH receptor (CRHR)^10^. Recently, urothelial expression of CRHR and UCN had been detected in an animal study^11^. In the FIC model, functional activation of CRHR by UCN was also shown to elicit ATP release^11^. However, the role of bladder CRH signaling in human IC/BPS is not well understood or investigated. The aim of the current study was to investigate the expression level of CRH in the bladder mucosa of patients with IC/BPS and potential clinical implication.

## Results

Of the 98 patients enrolled in the study, 51 had non-Hunner’s lesion IC/BPS (NHIC) and 23 had Hunner’s lesion (HIC), and 24 were control patients; bladder samples were obtained from all patients. The mean age of NHIC patients was 47.6 ± 11.9 years, which was significantly younger than the age of HIC patients and control subjects (59.9 ± 10.0 and 57.0 ± 12.8 years, respectively, p < 0.001). Table 1 lists the clinical symptoms scores and urodynamic parameters in patients with IC/BPS. The HIC patients had significantly higher ICPI (Interstitial Cystitis Problem Index), ICSI (Interstitial Cystitis Symptom Index), OSS (O’Leary-Sant symptom scores), and VAS (visual analog scale) pain scores than the NHIC patients. The MBC (maximal bladder capacity) and CBC (cystometric bladder capacity) were also significantly smaller in patients with HIC than in patients with NHIC. A total of 20 of the 98 patients had regularly used hypnotic medications (2 in the control subjects, 7 in the HIC, and 11 in the NHIC patients).

**Table 1:** Clinical symptoms scores and urodynamic parameters in HIC and NHIC patients.

Characteristic	HIC (n = 23)	NHIC (n = 51)	P-value
Age (years)	59.9 ± 10.0	47.6 ± 11.9	<0.001
ICSI	16.3 ± 4.1	12.2 ± 3.6	<0.001
ICPI	14.1 ± 2.8	11.5 ± 3.8	0.01
OSS	30.4 ± 6.7	23.4 ± 7.6	0.002
VAS	7.8 ± 2.3	4.7 ± 2.8	<0.001
CBC (ml)	189.7 ± 73.2	287.3 ± 75.9	<0.001
MBC (ml)	504.6 ± 166.1	635.1 ± 131.3	0.001

HIC: IC/BPS patients with Hunner’ lesion; NHIC: IC/BPS patients without Hunner’s lesion; ICSI: Interstitial Cystitis Symptom Index; ICPI: Interstitial Cystitis Problem Index; OSS: O’Leary-Sant symptom scores, VAS: visual analog scale; CBC: cystometric bladder capacity; MBC: maximal bladder capacity.

**Figure 1:**
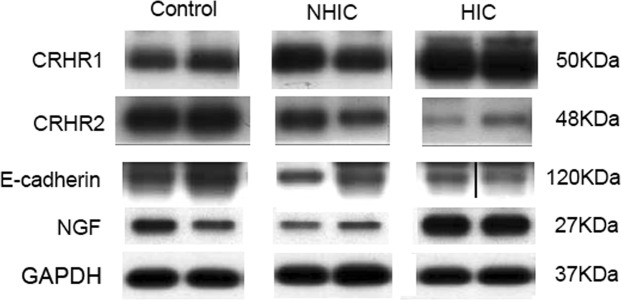
The representative western blotting figures of the bladder targets. The bands were cut from patients with HIC, NHIC and control subjects (from different experiments). CRHR1, CRHR2, NGF, E-cadherin were detected in bladder biopsy specimens, whereas UCN1 and UCN2 were not clearly expressed. Their corresponding full-length blots are presented in Supplementary Figs. 1–4.

**Figure 2:**
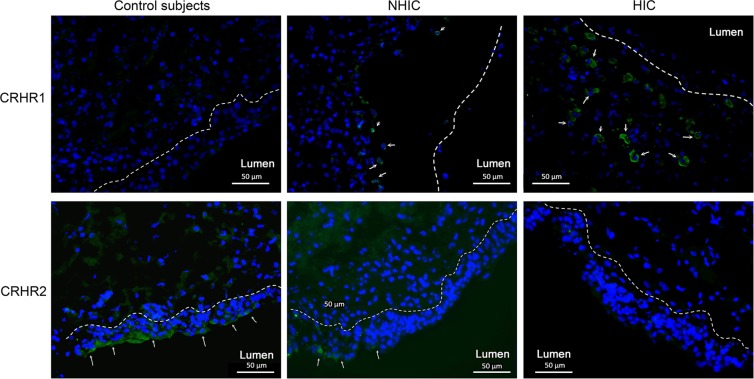
The immunofluorescence staining of CRHR1 and CRHR2 in the bladder of control, NHIC, and NHIC patients. The immunochemical staining revealed expression of CRHR1 in bladder submucosa but not in uroepithelial cells (white arrow). In contrast, CRHR2 expression was found mainly in uroepithelial cells (white arrow).

**Figure 3:**
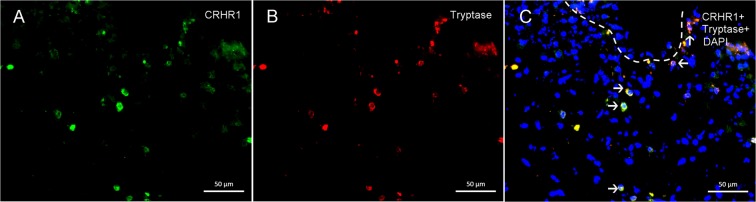
The double immunochemical staining of CRHR1 and tryptase in the bladder of IC/BPS patients. (**A**) CRHR1; (**B**) typtase; (**C**) merge figure for CRHR1, tryptase and DAPI. It showed the co-expression of CRHR1 and tryptase in the IC/BPS lamina propria (white arrow).

A total of 98 bladder samples were analyzed with western blot (control = 24, NHIC = 84, HIC = 23). CRHR1, CRHR2, NGF, and E-cadherin expressions in IC/BPS bladders were detected by western blotting, and the representative figure was showed in Fig. 1. The full-length blots are presented in Supplementary Figs. 1–5. The UCN 1 and UCN2 expression could not be clearly detected in control or IC/BPS bladder samples. In the immunohistochemistry staining, a total of 22 bladder samples were processed for CRHR1 and CRHR2 (control = 3, NHIC = 9, HIC = 10). The representative figures of CRHR1 and CRHR2 in the immunochemical staining were showed in Fig. 2. It revealed expression of CRHR1 in bladder submucosa but not in uroepithelial cells (Fig. 2). In contrast, the CRHR2 expression was mainly found in uroepithelial cells. The double immunochemucal staining revealed co-expression of CRHR1 and tryptase in the IC/BPS bladder lamina propria, this finding suggested the location of CRHR1 expression was on the activated mast cells (Fig. 3).

**Figure 4:**
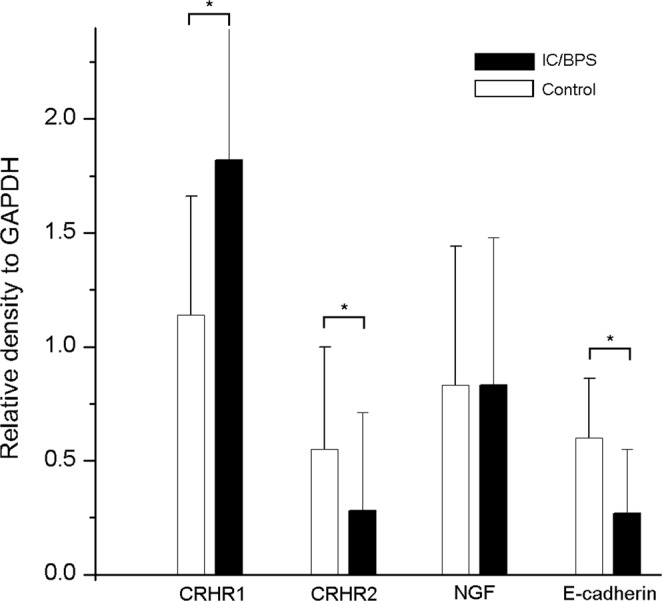
The total IC/BPS patients and control subjects bladder western blot results for CRHR1, CRHR2, NGF and E-cadherine. Statistical significance was tested with independent-T test. *p < 0.05.

**Figure 5:**
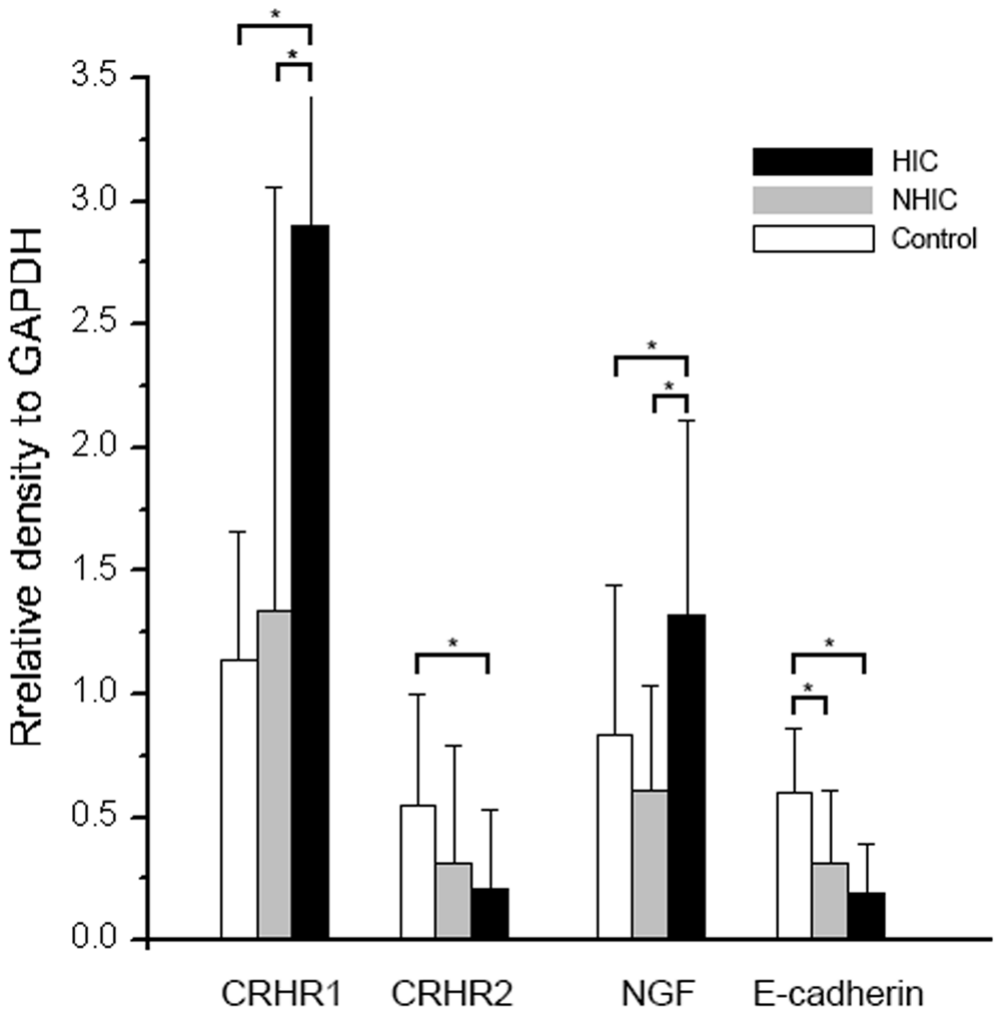
The HIC, NHIC and control subjects bladder western blot results for CRHR1, CRHR2, NGF and E-cadherine. Statistical significance was tested with one-way ANOVA followed by Scheffe posthoc test. *p < 0.05.

The western blotting quantification results are shown in Table 2. The bladder (including mucosa and submucosa) in total IC/BPS patients had significantly higher expression of CRHR1 than in the control subjects (Fig. 4, p = 0.004). In contrast, CRHR2 and E-cadherin expressions in the bladder of control subjects were significantly higher than that in patients with IC/BPS (p = 0.011 and <0.001, respectively). Further analysis of patients with HIC, NHIC, and control subjects showed that the bladder in patients with HIC had significantly higher expressions of CRHR1 and NGF (Fig. 5, both p < 0.001) while the bladder CRHR2 and E-cadherin expression was significantly lower (p = 0.03 and <0.001, respectively). The level of CRHR1 and CRHR2 expression in bladder did not have significant difference between the patient with or without using hypnotic medications.

**Table 2:** Bladder stress–associated receptors and neurotrophin expression in patients with IC/BPS and control patients.

	(A) Controls (N = 24)	(B) NHIC (N = 51)	(C) HIC (N = 23)	Total IC/BPS (N = 74)	P value*	P value**	Post hoc
CRHR1	1.14 ± 0.52	1.34 ± 1.72	2.90 ± 1.43	1.82 ± 1.78	0.004	<0.001	A vs B = 0.89 A vs C < 0.001 B vs C < 0.001
CRHR2	0.55 ± 0.45	0.31 ± 0.48	0.21 ± 0.32	0.28 ± 0.43	0.01	0.03	A vs B = 0.10 A vs C = 0.04 B vs C = 0.67
NGF	0.83 ± 0.61	0.61 ± 0.42	1.32 ± 0.79	0.83 ± 0.65	0.99	<0.001	A vs B = 0.29 A vs C = 0.02 B vs C < 0.001
E-cadherin	0.60 ± 0.26	0.31 ± 0.30	0.19 ± 0.20	0.27 ± 0.28	<0.001	<0.001	A vs B < 0.001 A vs C < 0.001 B vs C = 0.20

HIC: IC/BPS patients with Hunner’s lesion; NHIC: IC/BPS patients without Hunner’s lesion; CRHR: corticotropin-releasing hormone receptor; NGF: nerve growth factor.

The proteins expression levels were given by the relative intensity compared with GAPDH.

*P-value for Independent t-test comparing controls and total IC/BPS patients.

**P-value for ANOVA of control, non-ulcer, and ulcer IC/BPS patients.

The expressions of bladder CRHR1 and CRHR2 in patients with IC/BPS were negatively correlated (r = −0.200, p = 0.045). The bladder CRHR1 expression in IC/BPS patients was positively correlated with NGF (r = 0.422, p < 0.001) and negatively correlated with E-cadherin (r = −0.236, p < 0.001). In the association between bladder CRH signaling and the clinical symptoms of IC/BPS, CRHR2 was significantly negatively correlated with ICSI (r = −0.347, p = 0.010), ICPI (r = −0.396, p = 0.003), OSS (r = −0.413, p = 0.002), and VAS (r = −0.344, p = 0.011).

## Discussion

It is well established that psychological (as well as physical) stressors exacerbate the symptoms of IC/BPS. A significant relationship has been observed between stress and urinary urgency^6^. Various animal stress models (water avoidance, restraint) can result in several clinical and functional features similar to those of humans with IC/BPS^12^. However, human bladder tissues are rarely used to investigate the role of stress-response receptors in the pathogenetic mechanisms of IC/BPS. To our knowledge, our study is the first to investigate the expression of the stress-response receptors CRHR in the human bladder. Bladder mucosa CRHR1 was overexpressed in patients with IC/BPS while CRHR2 expression was significantly decreased. The CRHR2 expression mainly located in the uroepithelial cells, and the location CRHR1 expressions were mainly in the lamina propria. Co-expression of CRHR1 and tryptase was also noted in the double immunochecmical staining, and the location of CRHR1 expression should be in the mast cells. The expression of CRHR2 was also significantly correlated with clinical symptoms in IC/BPS patients. Thus, our study not only revealed that CRHR is expressed in the human bladder, but also that the levels of these receptors are altered and may contribute to sensory dysfunction in human IC/BPS.

The use of psychological stress in animals to investigate the features of IC/BPS can be traced back to 1997. Theoharides *et al*. used acute nontraumatic immobilization stress in rats and found increased activation of bladder mast cells in rats with stress versus the controls^13^. They further show decreased bladder mast cell degranulation after capsaicin or neurotensin antagonist injection and suggested acute stress might induce neurogenic inflammation in the rat bladder^14^. Studies have shown that chronic psychological stress induces urinary frequency, sustained bladder hyperalgesia, tactile hind paw allodynia, and suprapubic hyperalgesia. This model also reveals increased engagement of portions of the micturition circuit responsive to urgency. The WAS model has also been shown to exhibit alterations in the GI tract as a model for inflammatory bowel syndrome, which may be a comorbid condition associated with both LUTS and IC/BPS^7,8,15^. Though expression of CRHR in FIC mucosa was not changed from healthy control, there was a functional activation of CRHR in isolated urothelial cells, which is dysregulated in IC/BPS^11^. Our current human IC/BPS study not only revealed altered expression of CRHR in the bladder mucosa and submucosa, but also that expression of CRHR1 was significantly correlated with NGF and E-cadherin. These changes may impact urothelium barrier and sensory functions.

CRH plays a central role in the stress response by regulating behavioral and neuroendocrine responses to stress, but compelling evidence also reveals that CRH could exert a number of local biological actions^10^. Previous study had showed expression of CRHR1 in human skin mast cells, and CRH could induce mast cells degranulation and lead to secretion of vascular endothelial growth factor^16,17^. An animal study also revealed acute stress could trigger skin mast cell activation via CRH pathway, and suggested CRH might have important role of stress-induced neurogenic inflammation^18^. Our current study not only showed upregulation of CRHR1 in IC/BPS bladder, but also revealed the expression of CRHR1 was located in the activated mast cells. Our findings suggest the CRH pathway may be involved in the mechanism of neurogenic inflammation in IC/BPS bladder.

The CRH receptors and UCN are widely expressed in human intestinal tissue and have various physiological functions. UCN1 and CRHR1 can activate contractile activity in the duodenum through CRHR1 while inhibiting ileal contractions through CRHR2^19^. Recently, studies have established that CRH could act locally as a proinflammatory mediator, alter integrity of the intestinal epithelial cellular layer, dysregulate the tight junctions of the mucosa cells, and contribute to visceral pain in the gastrointestinal tract^20^. CRHR1 expression in the sigmoid colon in patients with ulcerative colitis was increased by 4.2 times compared to that in healthy subjects^21^. In another study, downregulation of CRHR2 in the sigmoid colon of patients with ulcerative colitis has also been observed^22^. In our study, the CRHR1 expression also was upregulated in the HIC bladders, while the CRHR2 expression was downregulated.

The changes in CRHR1 and CRHR2 seem to be more predominant in HIC bladder. We cannot yet explain the functional significance from these findings. However, activation of CRHR1 has been linked with visceral hyperalgesia while CRHR2 activation yields analgesia^23^. Thus it is likely that changes in CRHR expression in the bladder might play an important role in human IC/BPS, especially in patients with HIC. Amitriptyline, which is a tricyclic antidepressant, is considered as a safe and effective treatment for IC/BPS^21^. A previous animal study revealed the CRHR1 expression was decreased after long-term amitriptyline treatment^22^. Since our study revealed increased CRHR1 expression in bladder might have an impact on the IC/BPS pathogenesis, the therapeutic effect of amitriptyline in IC/BPS might be associated with CRH signaling system.

The main limitation of this study should be the problem of the antibody specify. Although we had purchased the most widely used antibodies, the antibody of NGF specificity was still a debatable issue. It might recognize both mature and immature NGF. Due to the small case number in this study, the results could be heavily weighted by a limited number of extreme values. Although we observed significant CRHR dysregulation in IC/BPS bladders, the detailed pathophysiology mechanism of CRH signaling in IC/BPS is still unclear. The association between CRHR expression and the other stress markers, such as urine or serum noradrenaline and cortisol levels, need to be determined in further study. In our experiment, the bladder specimens might be damaged by endoscopic biopsy, so the bladder targets expression might be affected by the damage. However, both the IC/BPS and control specimens were taken by endoscopic biopsy, the effect should be equivalent. Furthermore, the lack of CRHR1 in human uroepithelium versus that in previous murine and feline studies may reflect species differences or antibody specificity in the expression of CRHR subtypes. The CRHR expression change after treatment is important, but currently we do not have the data. Additional studies may be warranted to investigate the role of CRH in linking stress and functional bladder responses.

## Conclusion

Our findings reveal that patients with IC/BPS exhibit differences in CRHR expression within the urinary bladder urothelium and suburothelium. The CRHR1 expression in IC/BPS patients was significantly positively correlated with NGF and negatively correlated with E-cadherin. In contrast, CRHR2 expression was significantly correlated with clinical symptom scores, including those of the ICSI, ICPI, and VAS. While additional studies are needed to address the impact of CRHR signaling, the altered receptor expression suggests that CRH-related peptides within the bladder mucosa may play a role in IC/BPS pathophysiology.

## Materials and Methods

From 2012 to 2017, IC/BPS patients were admitted to our hospital for diagnostic cystoscopic hydrodistention and enrolled in this study. The diagnosis of IC/BPS was made on the basis of the American Urology Association Guidelines (an unpleasant sensation perceived to be related to the urinary bladder, associated with lower urinary tract symptoms of more than 6 weeks’ duration, in the absence of infection or other identifiable causes)^1^. IC/BPS patients with concurrent urological diseases such as bacterial cystitis, neurogenic voiding dysfunction, ketamine-related cystitis or urolithiasis were excluded. Patients who had undergone urological procedures in recent 6 months, such as cystoscopic hydrodistention or intravesical instillation of any therapeutic agent, were excluded. In addition, the patients with history of major depression, bipolar disorder and schizophrenia also would be excluded. This study was approved by the institutional review board and ethics committee of the Buddhist Tzu Chi General Hospital (IRB number 101-61). All patients voluntarily agreed to participate in this study and provided informed consent. In addition, patients with stress urinary incontinence who were admitted for anti-incontinence surgery were also enrolled as control subjects. All research activities were performed in accordance with the guidelines of the Declaration of Helsinki.

All of the IC/BPS patients underwent a comprehensive medical history investigation. Symptom severity was evaluated with questionnaires such as the ICSI, ICPI, OSS (ICSI + ICPI), and VAS. Urodynamic studies were performed to confirm the diagnosis and rule out the coexistence of other lower urinary tract diseases. The CBC was recorded. All patients underwent cystoscopic hydrodistention under general anesthesia at an intravesical pressure of 80 cm of water. The MBC during the procedure and grading of glomerulation hemorrhage after pressure release were recorded. The IC/BPS patients were classified into HIC and NHIC according to the cystoscopic finding of Hunner’s lesions before bladder distention. Random cold-cup biopsies of the posterior bladder wall including mucosa and submucosa in patients with NHIC were obtained after cystoscopic hydrodistention. The bladder biopsies of HIC were obtained at sites near the Hunner’s lesions with grossly intact mucosa. Bladder biopsies of the posterior wall were obtained from the control subjects. Endoscopic electrocauterization of the biopsy sites was also performed to prevent bleeding. Each specimen was 2 mm in diameter and contained mucosal and submucosal tissues but not detrusor muscle.

### Western blotting

The bladder biopsy specimens from the IC/BPS and control patients were homogenized in liquid nitrogen and then lysed for 10 min on ice using Hytra Tissue Protein Extraction Reagent (Hycell Biotechnology Inc., Maryland, USA). The extraction solution was supplemented with a protease inhibitor cocktail (Roche Diagnostics, Mannheim, Germany) and a phosphatase inhibitor cocktail (Roche Diagnostics). Proteins were separated by electrophoresis on 12% Tris-glycine gel. After gel electrophoresis, the protein blots were transferred to 0.2-µm PVDF membranes using 3% skim milk as blocking buffer for 1 hour, then adding CRHR1 (dilution 1:500), CRHR2 (dilution 1:1000, both CRHR1 and CRHR2 purchased from Abcam,Cambridge, UK, catalog number: ab59023 and ab150510), UCN1 (dilution 1:1000, from R&D Systems, Minnesota, USA. Catalog number: MAB7447), UCN2 (Mybiosource, California, US, Catalog number: MBS9127650), E-cadherin (dilution 1:5000, BD Biosciences, NJ, USA, Catalog number: 610181), or NGF (dilution 1:1000, from Abcam, Cambridge, UK, Catalog number: ab6199) as primary antibody and GAPDH (GeneTex, CA, USA, Catalog number: ab6199GTX100118) as positive control. The membranes were incubated for overnight at 4 °C; after incubation, they were washed with TBST 4 times each for 10 mins. The secondary antibody (goat anti-rabbit IgG-HRP; 1:5000, Santa Cruz Biotechnology, Santa Cruz, California, USA) was then applied. The membranes were finally probed with enhanced chemiluminescence reagent (ECL; Millipore Corporation, USA) and exposed to X-ray films. The scanned film after gel electrophoresis was quantified using a gel documentation system (Quantity One Version 4.6.2, Bio-Rad Laboratories, Hemel Hempstead, Hertfordshire, UK). GAPDH was used as normalizing protein for the quantification. All of the bladder samples from the all patients were analyzed with identically technique.

### Immunochemical staining

The immunochemical staining procedures were carried out by following our previously described experiment protocol^24^. The bladder specimens wer fixed in formaldehyde phosphate-buffered saline (pH, 7.4) solution for 1 hour, and then rinsed with phosphate-buffered saline containing 15% sucrose at 4 °C overnight. The specimens were embedded in OCT medium. The sample were cryosectioned with a thickness of 8 µm and were placed on Polysine glass slides. The sections were then fixed in acetone at −20 °C and incubated with blocking solution (BioGenex Laboratories, Santa Cruz, CA, USA). Subsequently, they were incubated overnight at 4 °C with the following primary antibodies for E-cadherin, NGF, CRHR1, CRHR2, UCN1, and UCN2. After rinsing the sections with 0.1% Tween 20 in PBS, the sections were stained with goat anti-rabbit IgG (conjugated to HRP) anti-mouse immunoglobulin/fluorescein isothiocyanate for 1 hour at room temperature. The sections were then counterstained with 4,6-diamidino-2-phenylindole (Sigma Aldrich). The negative control specimens were processed using the same procedure, except for the primary antibodies. Immunofluorescence-stained images were assessed using fluorescence microscopy and then processed with a digital imaging system (Carl Zeiss, Oberkochen, Germany). We also performed double immunochemical staining to detect the expression location of typtase (activate mast cell marker) and CHRH1. For double labelling the experiments, after the first incubation for CRHR1 antibody as described above, the bladder sections were incubated again with another primary antibody for typtase (dilution 1:1000, from Millopore, MA, USA) and with the anti-goat secondary antibody, following the same procedures.

### Statistical analysis

Data are expressed as mean ± standard deviation. The differences in clinical parameters between HIC and NHIC were analyzed using the independent T-test. The bladder stress-response receptors and neurotrophin expressions between different groups were analyzed using the independent T-test, one-way ANOVA, and the P trend test. A p-value <0.05 was considered significant. Pearson’s correlation coefficients were calculated to determine the correlations between clinical parameters and the results of western blotting. All calculations were performed using SPSS for Windows, version 16.0 (SPSS, Chicago, IL).

## Supplementary information

Supplementary information
