# Supplementary material for: Dysregulation of bladder corticotropin-releasing hormone receptor in the pathogenesis of human interstitial cystitis/bladder pain syndrome
Source: Sci Rep. 2019 Dec 16;9:19169. doi: 10.1038/s41598-019-55584-y (PMC6915757; doi:10.1038/s41598-019-55584-y)

Supplementary information

Title: Dysregulation of bladder corticotropin-releasing hormone receptor in the pathogenesis of human interstitial cystitis/bladder pain syndrome

List of authors: Jia-Fong Jhang, Lori A Birder, Yuan-Hong Jiang, Yung-Hsiang Hsu, Han-Chen Ho, Hann-Chorng Kuo

Supplementary Figure S1. Western blot membranes for bladder CRHR1

Test (2IC/BPS and 2 control subjects)

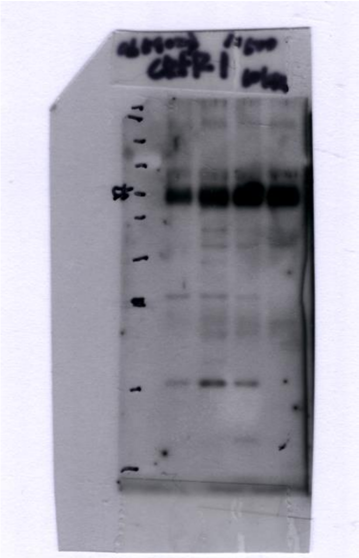

All subjects (target band cutting from different membranes, each band indicated CRHR1 expression in different IC/BPS patients or control subjects)

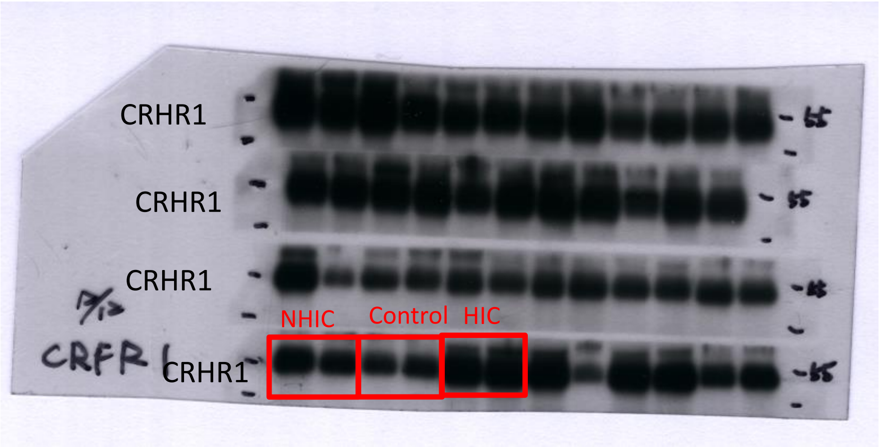

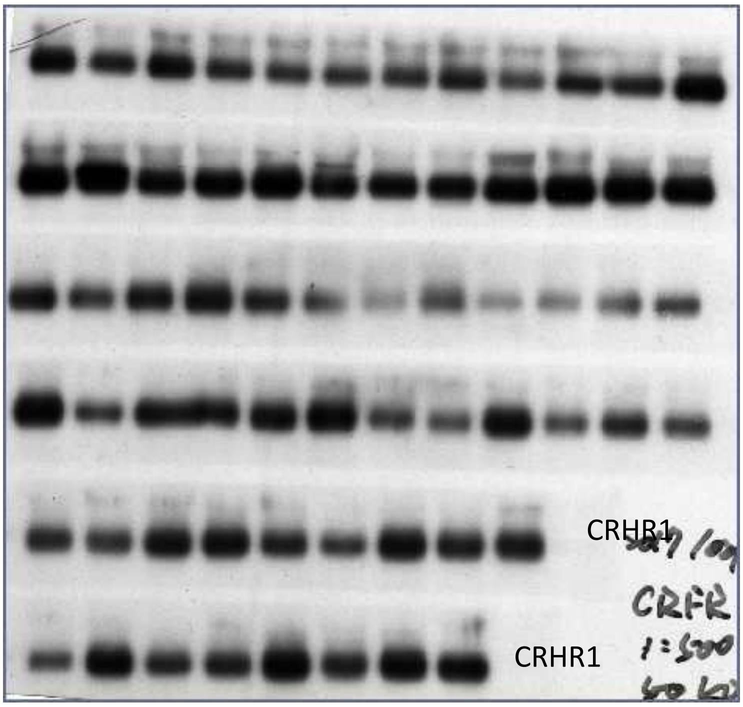

Supplementary Figure S2. Western blot membranes for bladder CRHR2

Test (2IC/BPS and 2 control subjects)

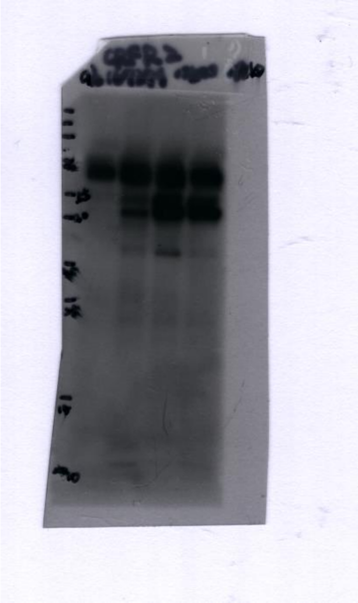

All subjects (target band cutting from different membranes, each band indicated CRHR2 expression in different IC/BPS patients or control subjects)

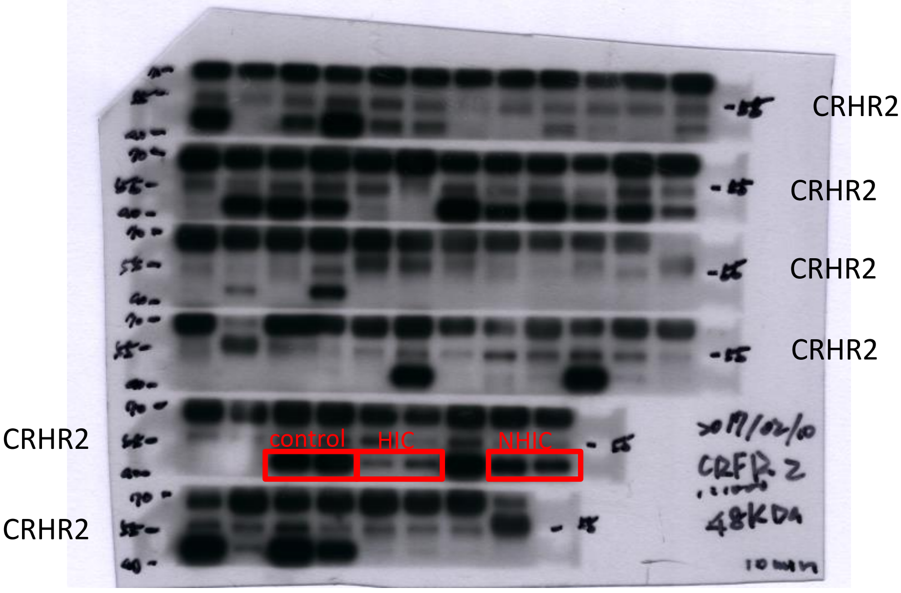

CRHR2

CRHR2

CRHR2

CRHR2

CRHR2

CRHR2

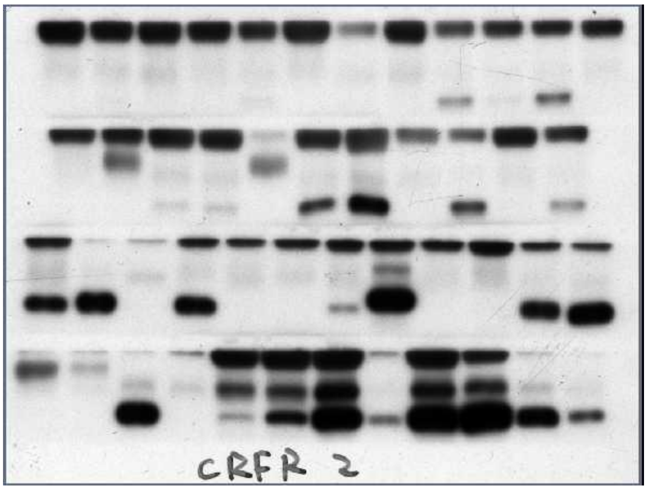

CRHR2

CRHR2

CRHR2

CRHR2

Supplementary Figure S3. Western blot membranes for bladder E-cadherin

Test (2IC/BPS and 2 control subjects)

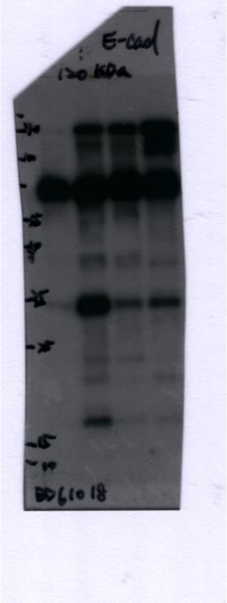

All subjects (target band cutting from different membranes, each band indicated E-cadherin expression in different IC/BPS patients or control subjects)

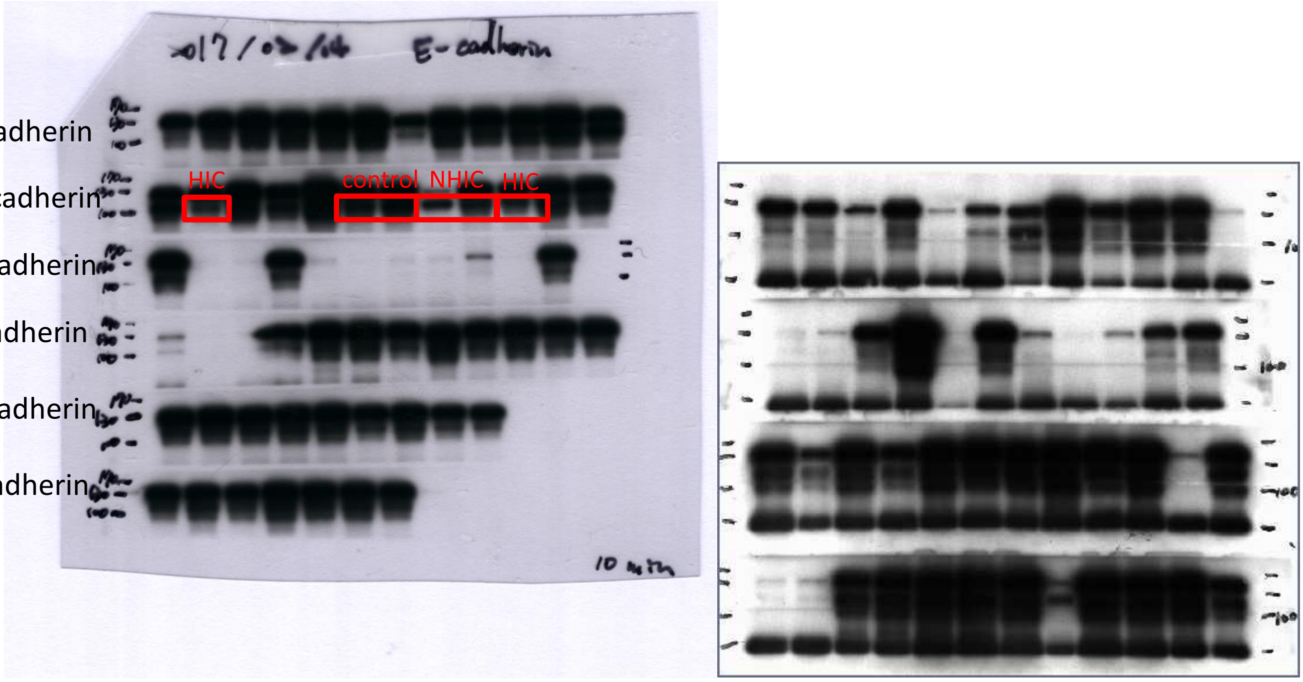

Supplementary Figure S4. Western blot membranes for bladder NGF

Test (2IC/BPS and 2 control subjects)

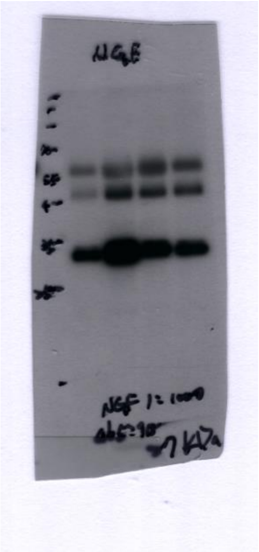

All subjects (target band cutting from different membranes, each band indicated NGF expression in different IC/BPS patients or control subjects)

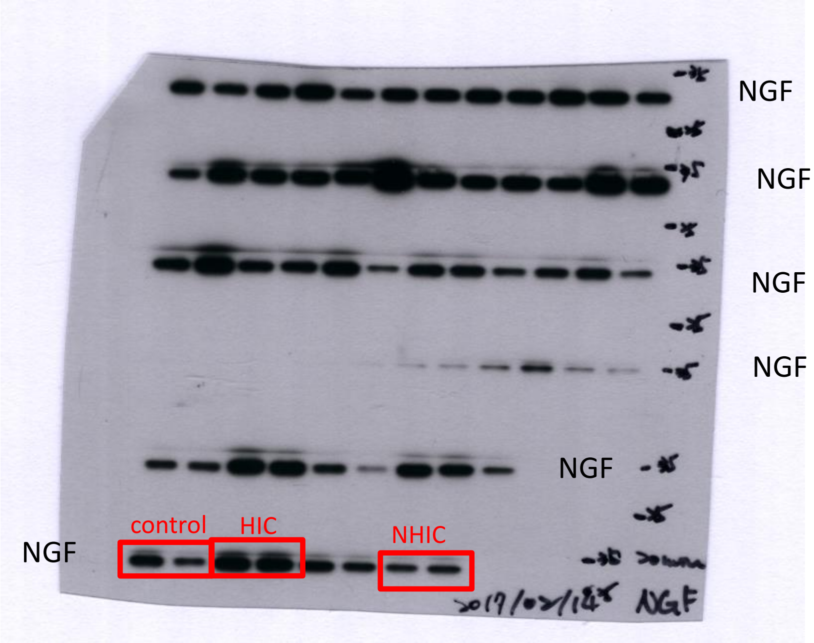

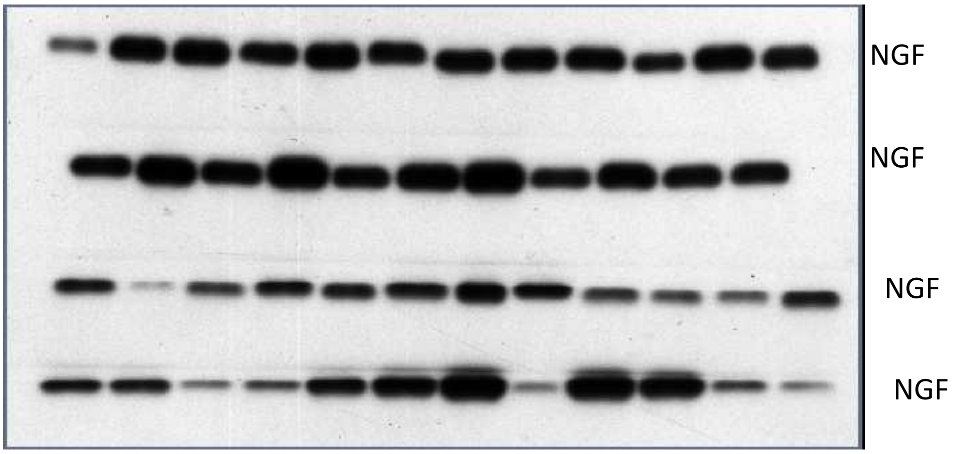

Supplementary Figure S5. Western blot membranes for bladder GAPDH

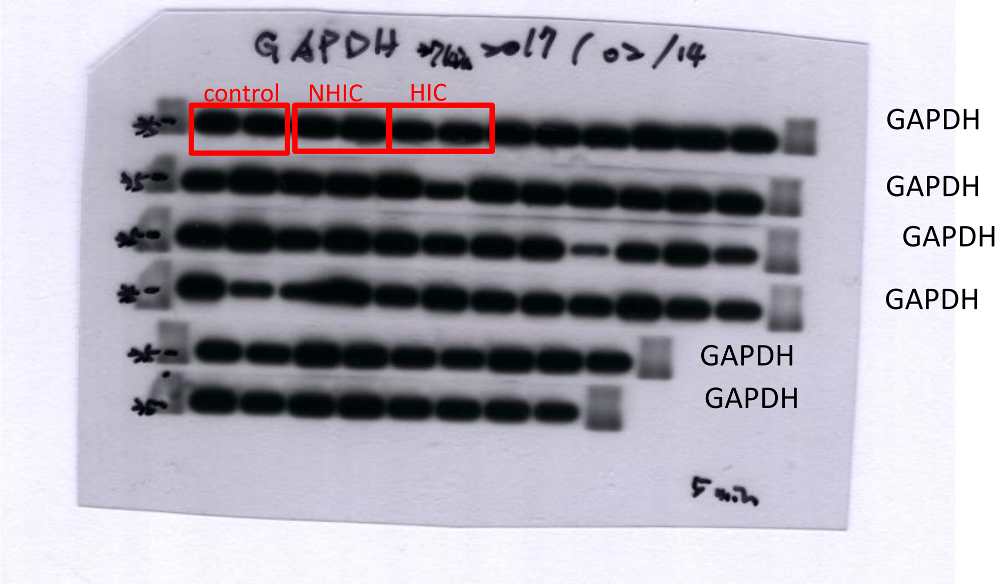

Supplement: Supplementary file 1 — Supplementary information [file 41598_2019_55584_MOESM1_ESM.pdf]
